# Progress in Surgery

**Published:** 1902-04-19

**Authors:** 


### Progress in Surgery.

**Fractures and Dislocations.**—The great value of massage in the treatment of sprains has been recognised for centuries, but as Sir William Bennett[1] points out, the similarity of the conditions in recent fractures and sprains has not often been noted, though it undoubtedly exists. A sprain may be followed by extravasation of blood, œdema, and involvement of tendons in the exudation in exactly the same way as in the case of fracture, and the treatment of these conditions in each case should be the same. It is not entirely correct to think that absolute immobility of the fragments is necessary after reduction has been accomplished, for under certain conditions of delayed union a little movement of the broken ends of the bone does more good than harm by stimulating the formation of callus. The dangers of thrombosis and embolism feared by some do not exist after massage more than in fractures treated by prolonged splinting. Massage must always be employed with skill and dexterity ; however it is not to be regarded as a substitute for treatment by splints on the one hand, or by operative measures on the other, but should be used as a rational adjunct to each. Mr. Howard Marsh[2] thinks that treatment by massage and movement should never be undertaken except under skilled advice. Movement might be employed in three forms : (1) forcible movement under an anæsthetic ; (2) passive movement subsequently ; (3) movements made by the patient against resistance. Exact diagnosis was of great importance, as sublocations and fractures were frequently overlooked. In recent dislocations daily passive movements instead of fixation should be carried out after reduction. Before movement is employed in injuries to joints, it should be determined whether the lesion was really within the joint or whether it concerned the surroundings. If movement within a limited range were free and normal then the injury was probably not within the joint but in the parts around. While recognising that early massage and ordinary methods of treatment, followed by an early recourse to movement, are all that is required in the majority of simple fractures to effect a good result in the function of the limb, Mr. J. Poland[3] admits that in many cases the disability is undoubtedly very great, and often permanent. Severe displacements of the fragments in so-called simple (or closed) fracture and cases in which it is quite impossible by manipulation to replace exactly the fragments in their original position are best treated by open operation. According to Mr. Golding Bird[4] the crux of the whole question of operating in simple fractures is, What is the proper test by which we are to judge of the efficiency of any line of treatment ?

Does it follow that because some irregularity of outline of the fracture remains after non-operative treatment, therefore this has been ineffectual; and that the surgeon is open to the charge of not having done the best for his patient? Patient and surgeon answer these two questions from different points of view. The patient nowadays tests his "cure" and the care his surgeon has taken—by the process of skiagraphy—quite apart from testing the efficiency of the limb, and because he *sees* his fracture still, and even a certain amount of bone displacement, meditates an action for malpraxis, although in length, axial line, and efficiency his leg may leave nothing to be desired. Whatever aid skiagraphy may afford, it must not be the sole test of the surgeon's work, nor indicate the standard up to which operative procedures shall be carried. A trivial irregularity of the line of union from the surface may seem by skiagraphy in the depths of the tissues to be an ugly looking fracture. The public must now learn that the skill of the surgeon lies not in trying to produce a good-looking bone scar but a sound limb, both to outward appearance and in utility. A writer in the *Medical Record*[5] lays down the general rule that in fractures, either simple or compound, when reduction is impossible by ordinary means, we must consider the advisability of the application of bone or ivory supports in the shape of collars, nails, or screws to maintain the ends of the bones in proper relation. He considers that ivory or bone nails or screws are probably the best forms to employ, and that it will, as a rule, not often be necessary to make use of the larger collar or intra-medullary cylinder. Mr. A. H. Tubby[6] makes a necessary distinction between delayed union and non-union, for the two are not identical; a fracture may be a long time joining, yet may ultimately do so, and thus serve as an example of delayed union. Doubtless many weakly patients with fractured limbs are kept too long in bed, and delay in union arises from this cause, for if the limb be properly fixed in plaster of Paris, and the patient be removed into fresh air, proper union rapidly sets in. The boundary line between delayed union and non-union is difficult to be drawn, but, fortunately, non-union is of rare occurrence—perhaps 0·2 to 0·8 per cent. of all fractures. The failure of the healing process occurs in certain situations as follows in order of frequency: the upper third of the femur; middle of the humerus; oblique fractures of the tibia; fractures of both bones of the forearm, and the lower jaw. Tubby condemns the text-book practices for non-union—such as injection of irritating fluids, acupuncture, introduction of setons and of foreign bodies, and electrolysis, also the rubbing of the ends of the fragments together and subcutaneous section of fibrous callus. Under asepticism refreshment of the ends of the fragments should nowadays be carried out, the fragments fastened together by wire or screws, or by Senn's bone ferule, or transplantation of bone may be performed. Mr. John Poland[7] has given an exhaustive though concise account of traumatic separation of the epiphyses. Formerly it was thought that infancy was the usual time for these injuries to take place, but later experience has shown that the most common period for separation to occur is between 11 and 18 years of age. He has proved that there are particular periods during the first two decades of life in which each epiphysis is more likely to be detached by violence than at other times. This fact is due to the anatomical conditions of the conjugal region of the ends of the bones at these periods. There is an enormous preponderance in number of separations in the male over those in the female sex, and the epiphysis of the upper are more often involved than those of the lower extremity. Practical experience has shown that the various epiphyses of the lower end of the humerus, taken collectively, are most frequently separated. Of the single epiphyses the order is as follows:—Upper epiphysis of humerus, lower epiphysis of radius, lower epiphysis of femur, lower epiphysis of tibia. In order of frequency, according to pathological specimens, is, however, different, viz.: (1) Lower epiphysis of femur; (2) lower epiphysis of radius; (3) upper epiphysis of humerus; (4) lower epiphysis of humerus; (5) upper epiphysis of tibia; (6) lower epiphysis of tibia. The first two on the latter list are injuries too prone to be accompanied by other severe lesions, terminating in death and verification by an autopsy.

[1] Med. Rec., N.Y., Oct. 26, 1901, and Practitioner, Aug., 1901. [2] Brit Med. Jour., Oct. 19, 1901., p. 1,151. [3] Clinical Jour., Aug. 7, 1901, p. 256. [4] Practitioner, Aug., 1901, p. 180. [5] Med. Rec., N.Y., Sept. 21, 1901, p. 458. [6] Brit. Med. Jour., Dec. 7, 1901, p. 1,649. [7] Practitioner, Sept., 1901, p. 275.

